# Prevalence and correlates of herbal medicine use among type 2 diabetic patients in Teaching Hospital in Ethiopia: a cross-sectional study

**DOI:** 10.1186/s12906-018-2147-3

**Published:** 2018-03-09

**Authors:** Abebe Basazn Mekuria, Sewunet Admasu Belachew, Henok Getachew Tegegn, Dawit Simegnew Ali, Adeladlew Kassie Netere, Eskedar Lemlemu, Daniel Asfaw Erku

**Affiliations:** 1 Department of Pharmacology, School of Pharmacy, College of Medicine and Health Sciences, University of Gondar, P.O. Box: 196, Chechela Street, Lideta Subcity Kebele 16, Gondar, Ethiopia; 2 Department of Clinical Pharmacy, School of Pharmacy, University of Gondar, Chechela Street, Lideta Subcity Kebele 16, Gondar, Ethiopia; 3 Department of Pharmacology, Bule Hora University, Bule Hora, Ethiopia; 4 Department of Pharmaceutics and Social Pharmacy, College of Medicine and Health Sciences, University of Gondar, Chechela Street, Lideta Subcity Kebele 16, Gondar, Ethiopia; 5 Department of Sociology, Debre Markos University, Debre Markos, Ethiopia

**Keywords:** Herbal medicine, Diabetes, Gondar, Ethiopia

## Abstract

**Background:**

Type 2 Diabetes Mellitus (T2DM) patients are increasingly using herbal remedies due to the fact that sticking to the therapeutic regimens is becoming awkward. However, studies towards herbal medicine use by diabetic patients is scarce in Ethiopia. Therefore, the aim of the current study was to explore the prevalence and correlates of herbal medicine use with different sociodemographic variables among type 2 diabetes patients visiting the diabetic follow-up clinic of University of Gondar comprehensive specialized hospital (UOGCSH), Ethiopia.

**Methods:**

A hospital-based cross sectional study was employed on 387 T2DM patients visiting the diabetes illness follow-up care clinic of UOGCSH from October 1 to November 30, 2016. An interviewer-administered questionnaire regarding the demographic and disease characteristics as well as herbal medicine use was completed by the study subjects. Descriptive, univariate and multivariate logistic regression statistics were performed to determine prevalence and come up with correlates of herbal medicine use.

**Results:**

From 387 participants, 62% were reported to be herbal medicine users. The most prevalent herbal preparations used were Garlic (*Allium sativum L.)* (41.7%), Giesilla *(Caylusea abyssinica (fresen.)* (39.6%), Tinjute *(Otostegia integrifolia Benth)* (27.2%), and Kosso *(Hagenia abyssinicaa)* (26.9%). Most of herbal medicine users (87.1%) didn’t consult their physicians about their herbal medicine use. Families and friends (51.9%) were the frontline sources of information about herbal medicine followed by other DM patients who used herbal medicines (28.9%).

**Conclusions:**

The present study revealed a high rate of herbal medicine use along with a very low rate use disclosure to the health care professionals. Higher educational status, a family history of DM, duration of T2DM and presence of DM complications were identified to be strong predictors of herbal medicine use. From the stand point of high prevalence and low disclosure rate, it is imperative for health care providers to strongly consult patients regarding herbal medicine use.

**Electronic supplementary material:**

The online version of this article (10.1186/s12906-018-2147-3) contains supplementary material, which is available to authorized users.

## Background

Diabetes mellitus (DM) is a chronic devastating disease. Despite the recent advances in care and management, still precipitates considerable morbidity and long term complications [1]. It’s a progressing chronic illness with anticipated global prevalence of more than 3 million by the year 2030 [2]. In Ethiopia, the number of patients living with diabetes is increasing alarmingly with a national prevalence near to be 6.5% [3]. Although insulin therapy is considered to be the corner stone in managing DM, treating diabetes remained as a challenge requiring considerable commitment to a life-long regimen imposed by the disease. Due to the chronicity of the disease and the need for life long therapy, several DM patients look for other alternative managements like using traditional herbal remedies [4–12].

According to World Health Organization (WHO), traditional medicine is defined as “health practices, approaches, knowledge and beliefs incorporating plant, animal and mineral based medicines, spiritual therapies, manual techniques and exercises, applied singularly or in combination to treat, diagnose and prevent illnesses and maintain well-being” [13]. Among the traditional medicine practices, the use of herbal medicines is the most popular and used by the general population and patients with chronic illnesses such as DM [14]**.** Patients with diabetes may prefer to use herbal remedies over modern medicine for a number of reasons including; dissatisfaction with the conventional treatment, treatment related adverse effects and the perceived suitability of herbal remedies with patients’ values and spiritual beliefs [8, 10, 12]. Recent literature reviews showed that prevalence of herbal remedies usage and other complementary therapies among DM patients varied immensely across different countries ranging from 15 to 75% [4].

In Ethiopia, the use of traditional herbal medicine has not been only common rather it’s also culturally acknowledged [15]. Nevertheless, the use of traditional herbal medicine in the general population and patients with chronic medical conditions like cancer and hypertension was well studied across different regions of Ethiopia [15–17], literatures on the prevalence and correlates of herbal medicine use among Ethiopian diabetes patients is lacking. Therefore, the present study was aimed to investigate the prevalence and factors associated with herbal medicine use among T2DM patients who attended the diabetes illness follow-up care clinic of UOGCSH, Gondar, Ethiopia.

## Methods

### Study design and setting

An institutional based cross-sectional study was underwent on 387 T2DM patients who visited the diabetes illness follow-up care clinic of UOGCSH from October 1 to November 30, 2016. UOGCSH is located in Gondar town, northwestern Ethiopia, 738 km away from the capital city of Ethiopia. It is the only referral hospital in the area with multiple specialty clinics such as Diabetes illness follow-up care clinic, which currently provides free service for more than 10,000 DM patients on outpatient level.

### Sampling and recruitment strategies

All ≥18 years old adult patients diagnosed with T2DM for at least 1 year prior to recruitment were taken as our source populations, while those patients who visited diabetes illness follow-up care clinic of UOGCSH to check blood sugar and refill medications during the data collection period were considered as a study population. Single population proportion formula was used with the assumption of 95% confidence interval, 5% margin of error, 72.3% prevalence [15–17] of herbal medicine use and 5% contingency for possible non-respondents. Considering the above points, the final sample size was determined to be 408. As to UOGCSH statistics and information office record, an estimated number of 25 T2DM patients visit the diabetes illness follow-up care clinic every day for blood sugar checkup and medication refill. Thus, the total number of T2DM patients who will come to the clinic during the two-month study period was calculated. The number of participants to be interviewed per day during the 60 days of data collection was estimated to be 7.

Systematic random sampling technique was employed to recruit the study subjects. With this, the number of patient who visited the clinic every day were divided to the number of participants to be surveyed per day so that every fourth T2DM patients available at the diabetes illness follow-up care clinic during the 2-month data collection period were included. We chose the 2-month follow-up period for data collection to avoid duplication of the cases as patients return to the clinic every 2 months.

### Survey instrument

Five nurses were trained to carefully collect the data through interviewer-administered questionnaire. Those collectors were properly trained on the instrument and ways of approaching the patients. The questionnaire was prepared via appropriate modification of items in previously used instruments regarding herbal medicine use by T2DM patients [12, 16, 17] and items were thoroughly reviewed for relevance by all the authors. The questionnaire, first prepared in English, was then translated to Amharic language (local language) and back to English so as to ensure that the translated version gives the proper meaning. Furthermore, a pretest was done on 20 T2DM patients, who were not included in the final analysis, and relevant modifications were instituted to the tool before the commencement of the gross data collection. Generally, the questionnaire included two main parts. Part one includes questions about the socio-demographic variables such as age, sex, marital status, educational level, age at diagnosis, as well as and disease related characteristics such as duration of the disease, family history of DM, and presence of DM complications. The second section aimed at assessing herbal medicine use, information source and discussion with health care providers about herbal medicine use. The use of herbal medicine among respondents was assessed by a series of questions including use of herbal medicines, type of herbal medicine used, purpose of use, source of information and any untoward effects encountered while using herbal medicines. Study subjects were labeled as herbal medicine users if they have taken herbal medicine(s) via any route of administration. Routine meal preparations and those that are taken as nutrients (vitamin supplements) were excluded. The survey instrument, along with a cover letter, is provided in Additional file 1.

### Statistical analysis

The data collected were entered into and analyzed using Statistical Package for the Social Sciences (SPSS) software version 21.0 for Windows (SPSS Inc., Chicago, IL). Frequencies and percentages were used to express different variables. Pearson’s chi-square test was employed to compare baseline characteristics of herbal medicine users and non-users. Initially, Univariate analysis was done and variables with *p*-value less than 0.25 were further taken to the multivariate logistic regression analysis for proper adjustment with the possible confounders. Odds ratio (OR) with 95% confidence interval (95% CI) were clearly written along with corresponding *p*-value (*p* < 0.05) as cut off points for declaring statistical significance.

## Results

Out of 408 T2DM patients assumed for study participation, 387 completed the survey resulting in a 94.8% response rate. More than half of the patients (57.4%) were female with a mean age of 52.5 ± 12.6 years. Majority of the respondents were Orthodox Christians (54%) and married (68%). The socio-demographic and disease characteristics of study participants are summarized in Table 1.

**Table 1:** Patient characteristics and factors associated with herbal medicine use, Gondar, Ethiopia (*N* = 387)

Variables	Overall (n, %)	Herbal medicine use (n)		*p*-value	AOR (95% CI)
		Yes (*n* = 240)	No (*n* = 147)		
Sex				0.310	
Male	165 (42.6)	91	74		–
Female	222 (57.4)	149	73		–
Educational status				0.001*	
Primary	93 (24.1)	46	47		
Secondary	203 (52.4)	122	81		1.90 (0.79–4.92)
Tertiary	91 (23.5)	72	19		1.72 (1.18–5.12)
Marital status					
Unmarried	124 (32)	60	64	0.042*	1
Married	263 (68)	180	83		0.67 (0.29–1.71)
Average monthly Income					
< 150 USD	187 (48.3)	98	89	0.032*	
> 150 USD	200 (51.7)	142	58		0.52 (0.15–1.72)
Employment status				0.162	
Unemployed	267 (69)	175	92		
Employed	120 (31)	65	55		0.89 (0.51–1.51)
Religion				0.347	
Orthodox	209 (54)	170	39		–
Muslim	101 (26.1)	46	55		–
Protestant	43 (11.1)	14	29		–
Catholic	19 (4.9)	7	12		–
Others^a^	15 (3.87)	3	12		–
Mean age at diagnosis (in years)	52.5	50.1	54.1		
Duration of disease				0.001*	
< 6 years	228 (58.9)	168	60		–
> 6 year	159 (41.1)	72	87		1.51 (1.31–4.79)
Presence of DM complications					
No	158 (40.8)	63	95	0.020*	1
Yes	229 (59.2)	177	52		1.45 (1.02–6.05)
Family history of DM				0.001*	
No	148 (38.2)	50	98		1
Yes	239 (61.8)	190	49		3.12 (1.62–8.05)

*Abbreviation*: *USD* United States dollar

*significant association (*P*-value less than 0.05)

^a^ Jehovah witness, Adventist

Around 240 (62%) respondents claimed as they used traditional herbal medicine while 147 (38%) were found to be non-users. The most commonly used herbal preparations were Garlic *(Allium sativum L.)* (41.7%), Giesilla *(Caylusea abyssinica (fresen.)* (39.6%), Tinjute *(Otostegia integrifolia Benth)* (27.2%) and Kosso *(Hagenia abyssinicaa)* (26.9%). Plant-based traditional medicines relevant for the treatment and management of diabetes among respondents are summarized in Table 2. Table 3 describes the characteristics of herbal medicine use among respondents.

**Table 2:** Plant-based traditional medicines relevant for the treatment and management of diabetes among respondents, Gondar, Ethiopia (*N* = 387)

Nomenclature			Parts used	Potential side effects and toxicities
Scientific	English	Local name^a^		
*Moringa. stenopetala*	Moringa, cabbage-tree	Sheferaw	Leaves (Commonly grounded into powder for mixing)	Causes uterine contractions, Inhibits CYP3A4 (inhibits metabolism of anti-diabetic drugs in the meglitinide class), Chronic kidney disease, Hepatotoxicty [36, 37]
Hagenia Abyssinicaa	East African rosewood	Kosso	Flower and leaf extracts	Hepatotoxicity, Diarrhea, Gastritis, Optic atrophy [38, 39]
*Aloe vera* (ferox species)	Cape aloes, Aloe Vera	Eret	Gel extract, Leaves Rind Stem	Volume depletion, Hypoglycemia, Photosensitivity, Hepatotoxicity, Nephrotoxicity [40–44]
*Clausena anisataa*	Horse wood	Limche	Leaf, stem, and Root extracts	Heavy metal bio-accumulation (Iron, cadmium, manganese), Hypoglycemia, Gastritis [29, 45–47]
*Allium sativum* L.	Garlic	Nech shinkurt	Bulb taken with ‘injera’ before breakfast	Severe allergic reactions, changes in the menstrual cycle, nausea; sweating, hypoglycemia, interact with some medicines like HIV protease inhibitors (eg, saquinavir) [31, 32]
Otostegia integrifolia Benth	Abyssinian rose	Tinjute	The wood burnt to fumigate homes, the aroma from the smoke smelled	Good safety profile [26]
Caylusea abyssinica (fresen.)		Giesilla	Root chopped and mixed with cold water and drenched	Convulsions, coma, diarrhea and lacrimation [27]

^a^Amharic language

**Table 3:** Prevalence and characteristics of herbal medicine use in the study population, Gondar, Ethiopia (*N* = 387)

Variables	Frequency (%)
Herbal medicine use since diagnosis	
No	147 (38)
Yes	240 (62)
Reasons for herbal medicine use (*n* = 240) *(more than one option is possible)*	
Family, tradition or culture	74 (30.8)
Belief in advantages of herbal medicine	97 (40.4)
Herbal medicine is accessible and available	66 (27.5)
Treatment of diabetes and other medical problems	23 (9.6)
Dissatisfaction with conventional therapy	102 (42.5)
Others	15 (6.2)
Reasons for not using herbal medicine among non-users (*n* = 147) *(more than one option is possible)*	
Additional burden	62 (42.2)
Afraid of side effect	105 (74.4)
The doctor didn’t prescribe herbal medicine	81 (55.1)
Lack of belief in the benefits of herbal medicine	89 (60.5)
Discuss with HCPs about herbal medicine use (*n* = 239)	
No	208 (86.6)
Yes	31 (12.9)
Reason for not discussing with HCPs (*n* = 240)	
Anticipating negative response about herbal medicine use	158 (65.8)
Insufficient information of herbal medicine	20 (8.4)
It is not important for doctor to know about my herbal medicine use	62 (25.8)
Satisfaction with herbal medicine use (*n* = 240)	
Satisfied	103 (42.9)
Average	89 (37.1)
Dissatisfied	48 (20)

*Abbreviation*: *HCPs* Health care practitioners

**Fig. 1:**
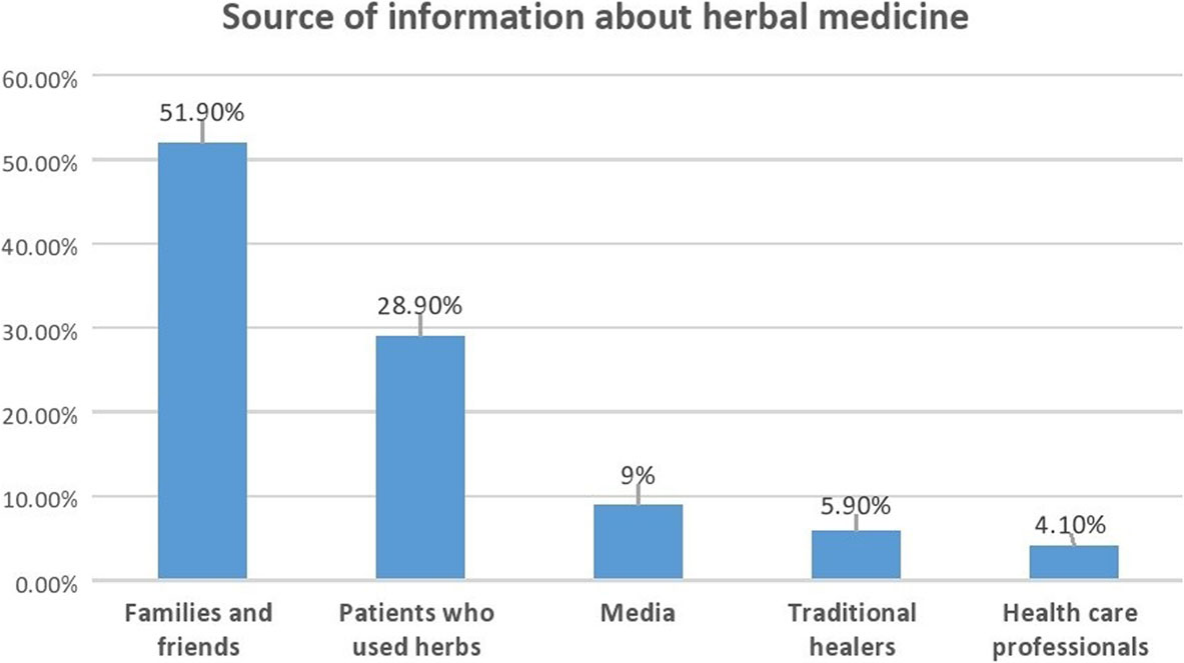
Source of herbal medicine use among users, Gondar, Ethiopia

According to the current study, families and friends (51.9%) were the frontline sources of information about herbal medicine followed by other DM patients who used herbal medicines (28.9%) (Fig. 1). Dissatisfaction with the modern therapy (28.9%) and beliefs in merits of herbal medicines (25.1%) were the key reasons for herbal medicine use, while afraid of side effect (40%) and lack of belief in the benefits of herbal medicines (23%) were reported to be the major reasons for not using herbal medicines among non-users. Large proportions (87.1%) of herbal medicine users did not inform their use with health care providers due to anticipation of negative reply towards herbal medicine use (65.8%). Details of characteristics of herbal medicine use is presented in Table 3.

As to the analysis performed, statistically significant differences in marital status, educational status, average monthly income, presence of DM complications, family history of DM and duration of DM were found between herbal medicine users and non-users (Table 1). Variables that were significantly associated with herbal medicine use in the bivariate analysis were further examined in multivariate regression. Accordingly, educational status, presence of DM complications, a family history and duration of DM remained to be significant in the multivariate analysis. The odds of herbal medicine use in patients with tertiary education were 1.72 times higher compared to patients with primary or lower education. The odds of herbal medicine use among patients with > 6 years duration of T2DM were 1.51 times higher compared to patients with < 6 years duration of T2DM. The odds of herbal medicine use among patients who develop DM complications were 1.45 times higher than in patients without DM complications and the odds of herbal medicine use among patients with a positive family history of the disease were 3.12 times higher than in patients without family history of the disease (Table 1).

## Discussion

Herbal medicine use is becoming a common practice both in developed and developing countries [5–12, 18, 19]. Patients with T2DM are increasingly using herbal remedies due to the resistance in sticking to the conventional therapies. In many Sub Saharan countries including Ethiopia, herbal medicine use is amplified by the presence of several traditional medicine practitioners. With this, the present study aimed at exploring the prevalence and correlates of herbal medicine use among T2DM patients who visited the diabetes illness follow-up care clinic of UOGCSH, Ethiopia.

According to the finding of present study, 62% of respondents used traditional herbal medicine. This result concurs with the study done in Tanzania [20]. The use of traditional herbal based medicine in Africa has been largely linked with cultural beliefs and advices from family as well as friends [21]. Patients usually use herbal remedies along with biomedical knowledge to improve the perceived value of their treatments [21]. The elevated prevalence of traditional herbal medicine use in our study can also be partially explained by the fact that Ethiopia is gifted with a rich and diverse flora that comprised a foundation for primary health care [22].

The traditional herbs recognized in this study are composed of numerous pharmacologically active compounds showing a variety of therapeutic effects. The most commonly claimed indications among T2DM patients include hypoglycemic and lipid lowering effect, anti-oxidative and anti-inflammatory effects. Furthermore, majority of herbs reported including *Hagenia abyssinica* [23], *Aloe vera* [24], *Clausean anistaa* [25], *Otostegia integrifolia Benth* [26] and *Caylusea abyssinica* [27] also had well-documented direct hypoglycemic effects or indirectly affecting glucose metabolism. Even though herbal products are shown to be effective in reducing the blood pressure [28], they contain toxic substances which could affect the health of the patient and render the conventional drugs ineffective. Despite their pertinent pharmacologic effects, majority of the identified herbs in this study also exhibit many untoward effects. For instance, *Clausena anisataa* (commonly known as Limche in Ethiopia) has tremendous potentially advantageous pharmacologic effects for T2DM patients including a hypoglycemic effect via inhibition of angiotensin-converting enzyme (ACE) [29]. However, it is also found to be linked with inhibition of reverse transcriptase inhibitors which have been used for the treatment of HIV/AIDS [30]. Similarly, *Aloe vera,* which is traditionally used for the treatment of burns and minor skin disorders, could end up with acute hepato-renal toxicity if ingested in huge amounts. Garlic, another important medicinal plant used by T2DM patients in our study, appears to exert numerous therapeutic effects through a number of mechanisms including inhibition of HMG-CoA reductase and platelet aggregation, increasing fibrinolytic activity, protecting against hematotoxins and stimulating insulin secretion. However, it has also been associated with serious allergic reactions, alterations in the menstrual cycle, nausea; sweating, hypoglycemia and interact with medicines such as HIV protease inhibitors (eg, saquinavir) [31, 32].

Hence, through understanding these important effects of herbs and promoting evidence-based discussions with patients, both conventional health care providers and local traditional medical practitioners may be capable ofIn our study, family members providing health care to T2DM patients in a more successful and patient-centered approach.

In multivariate analysis; higher educational status, a family history of DM, duration of T2DM and presence of DM complications were identified as a strong predictors of herbal medicine use. This finding supports a previous research outputs which reported an association of herbal medicine use with longer duration of diabetes and the presence of DM complications [4, 33]. Educational status was also shown to be the strong predictor of herbal medicine use which confirms the results of previous studies [10]. Patients with a higher educational status may be more likely to explore for other therapies and ways to muddle through with their disease state and treatment effects [34].

In our study, family members, friends and relatives were the common sources of information about herbal medicine. In contrast, medical practitioners were the least information source for herbal medicine use. Similar findings has been reported among other group of patients with chronic conditions in Ethiopia including cancer and hypertension, where the most prominent sources of information for herbal medicine and other types of complementary therapies were outside the medical scheme and included families, relatives and friends [16, 17]. To prevent the possible harm following herbal medicines use, health care professionals should emphasize safety issues to diabetes patients and make an effort to endow them with evidence-based information. Herbal medicine users in our study also rarely unveil their use to health care professionals [10], this is a burning issue which seeks particular attention. The major reason cited for not discussing with the doctor was due to forestalling negative response about herbal medicine use. This could be attributed to the fact that the general negative attitude of doctors to herbal medicine and their prominence on scientific evidence may discourage patients from sharing information about their herbal medicine use. The lack of communication between the health care provider and herbal medicine users may have a detrimental effect on patient health status as a result of toxic effect of herbs and their adverse interactions with the conventional treatments. They may also interfere negatively with glycemic control and cause untoward effects and additional complications [35].

### Strength and limitation

This survey highlights an area of research where there is lack of literature in the country. Yet, the survey has some limitations that should be taken into account while interpreting the results. As the study was a descriptive cross-sectional survey conducted in only one referral hospital, caution should be exercised when generalizing to other regions in Ethiopia. Our use of an interviewer-administered questionnaire, which depends on honesty and faith of the respondents, could also affect the responses as it may have been subjected to respondent or recall bias. Even with the above limitations, this survey has significant implications for improving the rational and evidence-based use of traditionally claimed medicinal herbs. A larger-scale and multi centered study that includes more diverse participants is needed to provide more accurate findings. In addition, we suggest future researchers to conduct similarly by using multiple samples for a single study population so as to come with more accurate and representative findings.

## Conclusions

The present study revealed a high rate of herbal medicine use along with a very low rate of disclosure to the health care providers. Commonly used herbs among T2DM patients were Garlic *(Allium sativum L.),* Giesilla *(Caylusea abyssinica (fresen.)*, Tinjute *(Otostegia integrifolia Benth)*, and Kosso *(Hagenia abyssinicaa)*. Patients mainly depend on families and friends as a source of information about herbal medicines. From the stand point of high prevalence and low disclosure rate, health care providers should often consult patients regarding herbal medicine use. Furthermore, the commonly used herbal preparations should also be further studied to confirm their efficacy and safety.

## Additional file

Additional file 1: Questionnaire used for the study. (DOCX 21 kb)

## Abbreviations

AOR: Adjusted odds ratio
CI: Confidence interval
OR: Odds ratio
SPSS: Statistical package for the social sciences
T2DM: Type 2 diabetes mellitus
UOGCSH: University of Gondar Comprehensive Specialized Hospital
USD: United States dollar
WHO: World Health Organization
