# Supplementary material for: Prevalence and correlates of herbal medicine use among type 2 diabetic patients in Teaching Hospital in Ethiopia: a cross-sectional study
Source: BMC Complement Altern Med. 2018 Mar 9;18:85. doi: 10.1186/s12906-018-2147-3 (PMC5845232; doi:10.1186/s12906-018-2147-3)
Supplement: Supplementary file 1 — Questionnaire used for the study. (DOCX 21 kb) [file 12906_2018_2147_MOESM1_ESM.docx]

**University of Gondar**

**College of Medicine and Health Sciences, School of pharmacy**

**Consent Information sheet**

My name is __________________________. I am here on behalf of Gondar University research group staffs. We are conducting a research on **‘Prevalence and factors associated with use of traditional herbal medicine among Type 2 diabetic patients in follow-up care at University of Gondar Teaching Referral Hospital, Ethiopia’** Their research project is approved by the Research Ethics committee of Gondar University. You are selected randomly to participate in this study. Your participation is purely based on your willingness. You have the right to choose not to take part in this study. If you choose to take part, you have the right to stop at any time. If you are willing to participate or refuse or decide to withdraw later, you will not be subjected to any ill-treatment.

If you agree to participate in the study, you will be asked to answer some questions about yourself and your family, your knowledge about CAM use. The interview with you will take about 20 minutes. The study will explore about the use and correlates of CAM. It can also provide baseline data for policy makers and other researchers for further improvements of CAM use. The information that you provide will be kept confidential by using only code numbers and locking the data.

Based on the understanding of the information I gave you, are you willing to participate in this study?

1. Yes
2. No

**Part I:** **Socio demographic and disease characteristics of respondents**

| **Sr. No** | **Questions** | **Choice Answers** |
| --- | --- | --- |
| 101 | Sex | 1. Male 2. Female |
| 102 | Age | in years: __________ |
| 104 | Marital status | 1. Single (not married, separated, widowed, divorced) 2. Married/living with a partner |
| 105 | Religion | 1. Orthodox 2. Muslim 3. Protestant 4. Catholic 5. Other, specify__________ |
| 106 | Occupation status | 1. Employed 2. Unemployed |
| 107 | Educational Status | 1. Illiterate 2. Primary education (1-8) 3. Secondary education (9-12) 4. Tertiary education (college and above) |
| 108 | Average monthly family income | in USD___________ |
| 109 | How long have you had diabetes | 1. <1 year 2. 1-2 years 3. 3-5 years 4. >6 years |
| 110 | Do you have a family history of diabetes? | 1. Yes 2. No |
| 111 | Do you have any of the following long term complications of diabetes (Peripheral Neuropathy (pins and needles or loss of feeling in the hands or feet), Kidney disease, eye and heart disease, Sexual dysfunction)? | 1. Yes 2. No |

**Part II: Herbal medicine use**

| 112 | Have you used herbal medicine since your diagnosis with T2DM? | 1. Yes 2. No |
| --- | --- | --- |
| 113 | If you have not used herbal medicine, why not? | 1. Lack of belief in the benefits of herbs 2. Afraid the side effect 3. Lack of availability 4. Didn’t get sick during gestation 5. Others, specify………… |
| 115 | Why have you used herbal medicines? (more than one option is possible) | 1. Family, tradition or culture 2. Belief in effectiveness of herbal medicines 3. Herbal medicines are cheap and accessible 4. Treatment of other medical problems 5. Safe in pregnancy 6. Others, specify………… |
| 116 | Are you using herbal medicines as alternative or as complementary to the conventional treatment? | 1. Alternative 2. Complementary 3. I don’t know |
| 117 | What type of herbal medicines have you used? More than one answer is possible | Please specify |
| 118 | For what purpose did you use those herbal medications? | Please specify |
| 118 | What is your source of information about herbal medicines? | 1. Families, friends and relatives 2. Media (internet, television, radio, book) 3. Health practitioner 4. Pregnant women who used herbal medicines 5. Others, specify_______________ |
| 120 | Have you had any untoward effects from herbal medicines use? | 1. Yes 2. No |
| 121 | Have you ever discussed using herbal medicines with your Health care provider? | 1. Yes 2. No |
| 122 | In general, how could you rate the advantage you get from using herbal medicines use? | 1. Satisfied 2. Average 3. Dissatisfied |
| 123 | Would you use herbal medicine again? | 1. Yes 2. No 3. undecided |

**Thank you for your participation!!!**
